# Risk stratification for predicting postoperative recurrence of gastric cancer by grade of venous invasion

**DOI:** 10.1186/s12876-023-02825-0

**Published:** 2023-05-30

**Authors:** Yasuo Imai, Yoshihiro Kurata, Masanori Ichinose

**Affiliations:** 1 Department of Diagnostic Pathology, Ota Memorial Hospital, SUBARU Health Insurance Society, 455-1 Oshima, Ota, 373-8585 Gunma Japan; 2 Department of Digestive Surgery, Shioya Hospital, International University of Health and Welfare, Tochigi, Japan; 3 Department of Frontier Surgery, Graduate School of Medicine, Chiba University, Chiba, Japan

**Keywords:** Gastric cancer, Stomach cancer, Venous invasion, EVG staining, Recurrence, Metastasis, Adjuvant chemotherapy, ACTS-GC, S-1, Differentiation grade

## Abstract

**Background:**

Venous invasion (VI) in pathological examination of surgically resected gastric cancer (GC) may predict postoperative recurrence, but there are no objective criteria for VI grading.

**Methods:**

157 GC patients (pathological stages I 82, II 34, and III 41) who underwent surgery with curative intent were analyzed. VI was graded in pathological examination by elastica van Gieson staining based on the number of VIs per glass slide as follows: v0, 0; v1, 1−3; v2, 4−6; and v3, ≥ 7. Filling-type invasion in veins with a minor axis of ≥ 1 mm increased the grade by 1. The association of VI grade with prognosis was statistically analyzed.

**Results:**

Recurrence increased with VI grade (v0 1.5%, v1 29.6%, v2 41.7%, v3 78.6%). VI grade as well as pathological (p) tumor, node, metastasis (TNM) stage was a significant recurrence predictor by the multivariate Cox analysis. VI grade was implicated in hematogenous and peritoneal recurrences independent of pTNM stage but not in nodal recurrence. GC was then divided into two tiers, without indication of adjuvant chemotherapy (AC) (pStage I, pT1 and pT3N0) and with AC indication (pStages remaining II/III), based on the ACTS-GC trial, which is common in Japan and East Asia. VI grade was a significant recurrence predictor in both tiers. v2/v3 revealed a significantly worse recurrence-free survival (RFS) than v0/v1 in GC without AC indication. v0/v1 exhibited RFS rate exceeding 95% even after 5 years but that of v2/v3 fell around 70% within one year postoperatively, suggesting that AC may be considered for this tier with v2/v3. GC with AC indication exhibited dismal RFS according to the VI grade. RFS rate fell below 80% within one year postoperatively when VI was positive, while recurrence was not observed in v0, which was, however, rare in this tier (10.9%). Differentiation grade did not significantly affect postoperative prognosis in both tiers.

**Conclusions:**

VI grade was a significant predictor of postoperative GC recurrence irrespective of the AC indication based on the ACTS-GC study and this VI grading system could be applied in future studies of adjuvant therapy in GC presently deemed without AC indication in Japan.

**Supplementary Information:**

The online version contains supplementary material available at 10.1186/s12876-023-02825-0.

## Background

Gastric cancer (GC), more than 90% of which is adenocarcinoma, is one of the most common and deadly neoplasm in the world. GC is subdivided into the gastroesophageal junction/cardia cancer and non-cardia cancer depending on its location. Its incidence and mortality have been decreasing in the past few decades because of the falling rates of non-cardia GC that is linked to a decline in Helicobacter pylori infection [1]. Nevertheless, GC is still responsible for estimated 1,089,103 new cases and 768,793 deaths in 2020 and ranks fifth for incidence and fourth for mortality globally [2]. The 5-year relative survival rates for GC have been reported to be 70% for the localized stage, 32% for the regional stage, 6% for the distant stage, and 32% for all stages combined [3]. Thus, when GC spreads outside of the stomach, its prognosis is poor.

Pathologists evaluate various pathological parameters, such as histological subtype, grade of differentiation, depth of tumor invasion, lymphovascular invasion, nodal metastasis, and resection margin status of resected GC specimens in the routine practice. Metastasis is caused by tumor cell spread via lymphatic, vein or by dissemination, and venous invasion (VI) is theoretically a risk factor for hematogenous metastasis. In the Union for International Cancer Control (UICC) tumor, node, metastasis (TNM) staging system (8th ed.) published in 2017, V1 and V2 are defined as microscopic and macroscopic VI, respectively, but VI is not implicated in the stage definition of the UICC TNM staging system [4]. Meanwhile, the latest Japanese Classification of Gastric Carcinoma (JCGC) (15th ed.) published in 2017 classified VI as V0 (none), V1a (mild), V1b (moderate), and V1c (severe) based on the pathologist’s subjectivity [5]. However, there are no such objective criteria for grading VI as enable prediction of postoperative recurrence. The purpose of this study was to determine the criteria in surgically resected GC without distant metastasis at the time of surgery. VI was evaluated by using an elastica van Gieson (EVG) staining, which is inexpensive and feasible at any facility worldwide.

## Methods

### Patients

Consecutive 226 patients who underwent resection of primary GC with curative-intent at International University of Health and Welfare, Shioya Hospital between May 2006 and June 2019 were analyzed. Inclusion and exclusion of patients were performed as had been explained in our previously published study [6]. Patients with gastroesophageal junction cancer (Siewert type II) [7], carcinoma in situ/high grade dysplasia, squamous cell carcinoma, and distant metastasis found prior to or at the time of surgery (clinical or pathological stage IV), and patients without nodal dissection were excluded. Patients with invasive cancers resected between 5 years before gastrectomy and 5 years after gastrectomy were also excluded, except for those with synchronous multiple GCs at gastrectomy. Patients with asynchronous invasive cancers that developed later than 5 years after gastrectomy were included and censored at the time of diagnosis of the new tumors. Clinicopathologic information as of June 2022 was obtained via the electronic chart system and patients without complete clinical information were excluded.

Patients’ follow up and adjuvant chemotherapy (AC) were performed as had been explained in our previously published study [6]. AC was initiated in eligible patients with pathological (p) TNM stages II and III within 4 to 6 weeks postoperatively. The regimens were principally either S-1 (Tegafur/gimeracil/oteracil) or paclitaxel in the case of S-1 intolerance for one year [8, 9] Schedule and doses were modified according to the patients’ performance status. Patients were followed up every three weeks during AC. Patients who did not receive AC were followed up monthly for the first one to two years according to the patients’ pTNM stages. Follow-up was then continued every 2 months until 5 years postoperatively or censored for social reasons. Blood test was performed every 2 months, and gastroscopy and contrast-enhanced computed tomography were performed every 6 months for the first year and yearly for 4 more years. No patients received adjuvant radiotherapy or chemoradiotherapy.

This study was conducted in accordance with the Declaration of Helsinki. The study protocol was approved by the ethical review board of the International University of Health and Welfare: 21-B-40 (1/19/2022).

#### Pathological examination

**Fig. 1:**
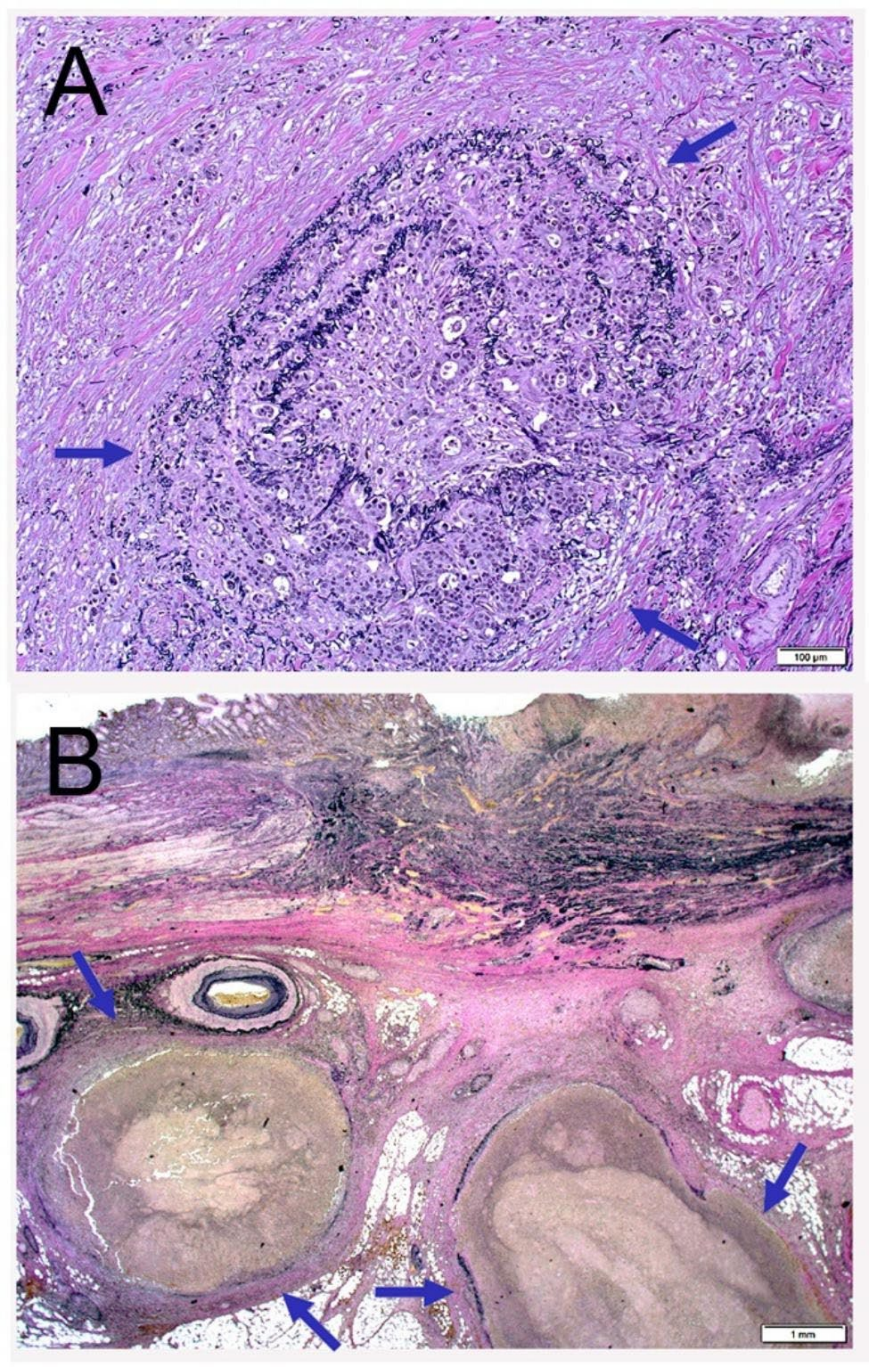
Representative histopathology of venous invasion. **a** Microscopic venous invasion. Cancer cells destroy and invade the circular elastic plate adjacent to the artery. **b** Filling type of venous invasion. Tumor emboli are found in the veins with a diameter greater than 1 mm

All surgical specimens were routinely processed for pathological diagnosis and pathological diagnosis was performed in the same way as had been explained in our previous studies by a single pathologist (Y. I.) with an experience of more than 30 years [6, 10]. Early cancer extending no more than submucosa at gross diagnosis was subjected to microscopic inspection of the whole tumor area. In advanced cancer invading no less than proper muscle, the maximal cut surface of the tumor, involving the transition between the tumor and normal mucosa, and the cut surface involving the deepest tumor penetration were microscopically inspected. Clinicopathologic classifications and stage groupings were performed based on the World Health Organization (WHO) classification of tumors of the stomach (5th ed.) and the UICC TNM staging system (8th ed.) [4, 11]. Histological classification was based on the predominant histologic pattern of the carcinoma [12, 13] and cancer stromal volume and infiltration pattern were classified based on the JCGC (3rd English ed.) [14]. EVG staining was performed in two or more, if necessary, sections that included the deepest tumor penetration and the area of transition between the tumor and normal mucosa. Each section usually contained approximately 2 to 5 cm^2^ of tissue per glass slide. When tumor cells invaded or were located in the tubular structure formed by an elastic plate adjacent to the artery, which means an adventitia of the vein, VI was diagnosed (Fig. 1A). VI in each section was graded according to the number of VI irrespective of the location: v0 (none), no venous invasion; v1 (mild), 1 to 3 venous invasions per glass slide; v2 (moderate), 4 to 6 venous invasions per glass slide; and v3 (severe), ≥ 7 venous invasions per glass slide. The VI grade in each case was based on the maximal grade in the investigated sections. When filling-type VI, in which tumor cells filled the vascular lumen, was found in a macroscopically identifiable vein with a minor axis of ≥ 1 mm, the grade of v1 or v2 was raised by 1 (Fig. 1B). The most predominant histological subtypes, the deepest tumor invasion, the highest VI grade, and the highest pTNM stage were recorded when they had multiple synchronous GCs.

### Statistical analysis

Statistical analysis was performed in the same way as had been explained in our previous studies [6, 10]. Categorical parameters between two patient cohorts and associations between two categorical variables were compared using Fisher’s exact test in the case of 2 × 2 cross tabulations or the chi-square test with or without Yates’ correction as appropriate in the case of m × n cross tabulations. Age, depth of tumor invasion, VI grade, nodal metastasis, resection margin status, and pTNM stage were compared using the Mann–Whitney *U*-test (Table S1). Associations between clinicopathologic parameters and recurrence/metastasis, which will be collectively referred to as recurrence hereinafter, were analyzed by the univariate Cox regression analysis. Multivariate Cox regression analysis was performed by forced entry method on selected parameters with *p* values < 0.10 by the univariate analysis. Survival curve analysis was performed using the Kaplan−Meier method with the log-rank test. *p* values < 0.05 were considered significant. Statistical analyses were performed using IBM SPSS Statistics 20 (IBM Corp, Armonk, NY, USA).

## Results

### Clinicopathologic features and associations of the VI grade with recurrence

A total of 157 patients, comprising 82 pStage I, 34 pStage II, and 41 pStage III, were analyzed (Table 1). Patients consisted of 111 males and 46 females, aged 42–96 (median, 72). The follow-up periods from surgery to cancer-related death or censoring were 22 to 5,479 days (median: 1,846 days). Recurrence was observed in a total of 38 (24.2%) patients, comprising 3 with pStage I, 10 with pStage II, and 25 with pStage III. Recurrence sites were residual stomach in 2 (1.3%) patients, non-regional lymph node in 11 (7.0%), peritoneum in 14 (8.9%), liver in 11 (7.0%), lung in 2 (1.3%), pancreas in 2 (1.3%), pleura in 2 (1.3%), bone in 2 (1.3%), duodenum in 1 (0.6%), portal vein in 1 (0.6%), and unknown site in 6 (3.8%) patients. Hematogenous metastasis was observed in 17 (10.8%) patients. There were significant differences in age, macroscopic type, depth of tumor invasion, cancer stromal volume, VI grade, lymphatic invasion, nodal metastasis, resection margin status, pTNM stage, and AC between patients with and without recurrence (Table 1).

**Table 1:** Clinicopathologic characteristics of gastric cancer

Parameters	Total (n = 157)	Recurrence		*p value*
		Yes (n = 38)	No (n = 119)	
Age				
Median (range)	72 (42–96)	76 (42–96)	70 (42–92)	0.004
Sex				
Male/Female	111/46	30/8	81/38	0.226
Location				
Upper/Middle/Lower/extending in 2 or 3 regions	32/23/91/11	6/4/23/5	26/19/68/6	0.798
Surgery				
DG/TG/others	104/50/3	24/13/1	80/37/2	0.692
Lymph node dissection				
D1/D1+/D2	13/68/76	3/14/21	10/54/55	0.732
Synchronous multiple GCs				
Yes/No	17/140	4/34	13/106	1.000
Macroscopic type				
0/1/2/3/4/5	78/10/25/17/17/10	3/4/11/7/10/3	75/6/14/10/7/7	< 0.001
Histology				
pap tub1/tub2/muc/por sig nec	41/33/1/82	6/5/1/26	35/28/0/56	0.167
Depth of tumor invasion				
pT1/pT2/pT3/pT4	78/15/42/22	3/3/15/17	75/12/27/5	< 0.001
Cancer stromal volume				
med /int/sci/not described	5/72/38/42	0/18/19/1	5/54/19/41	0.027
Tumor infiltration pattern				
INFa/INFb/INFc/not described	4/70/54/29	1/16/21/0	3/54/33/29	0.220
VI grade				
v0/v1/v2/v3	65/54/24/14	1/16/10/11	64/38/14/3	< 0.001
Lymphatic invasion				
Yes/No	82/75	35/3	47/72	< 0.001
Nodal metastasis				
pN0/pN1/pN2/pN3	91/20/19/27	4/8/9/17	87/12/10/10	< 0.001
Resection margin status				
R0/R1/R2	149/7/1	32/5/1	117/2/0	0.001
pTNM stage				
I/II/III	82/34/41	3/10/25	79/24/16	< 0.001
Neoadjuvant chemotherapy				
Yes/No/unknown	2/140/15	1/35/2	1/105/13	0.444
Adjuvant chemotherapy				
Yes/No/unknown	52/87/18	23/10/5	29/77/13	< 0.001

*DG* distal gastrectomy, *TG* total gastrectomy, *GC* gastric cancer, *pap* papillary adenocarcinoma, *tub1* well-differentiated tubular adenocarcinoma, *tub2* moderately differentiated tubular adenocarcinoma, *muc* mucinous adenocarcinoma, *por* poorly differentiated adenocarcinoma, either solid or poorly cohesive type, *sig* signet-ring cell carcinoma, *nec* neuroendocrine carcinoma, *VI* venous invasion, p*TNM* pathological tumor node metastasis, *v0* no venous invasion, *v1* 1–3 invasions/slide, *v2* 4–6 invasions/slide, *v3* no less than 7/slide. Filling type of venous invasion in macroscopically identifiable vein with a minor axis of ≥ 1 mm raised the grade of v1 or v2 by 1

**Fig. 2:**
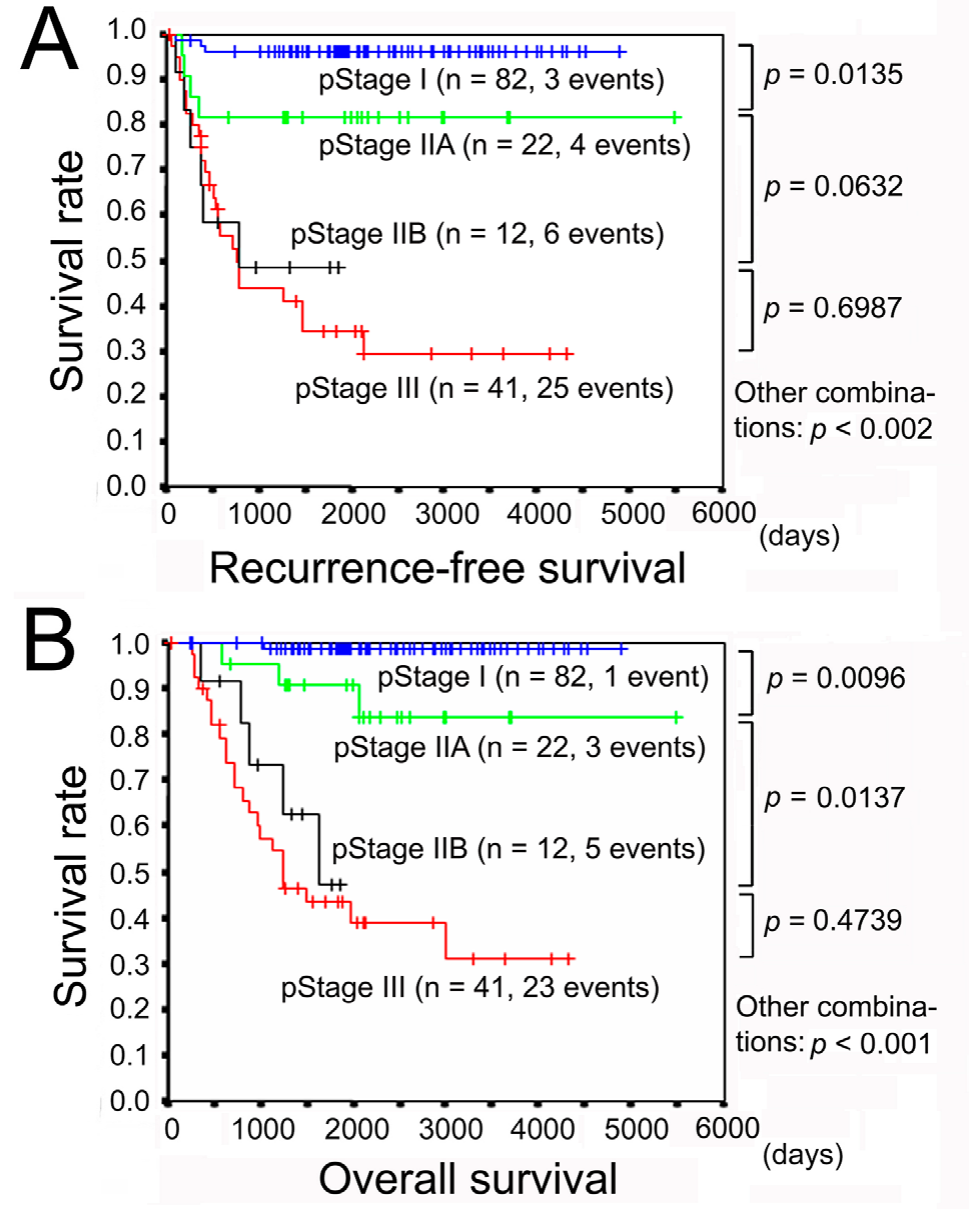
Postoperative prognoses of gastric cancer. **a** Recurrence-free survival. **b** Disease-specific overall survival

The recurrence free survival (RFS) and disease-specific overall survival (OS) deteriorated according to the pTNM stage (Fig. 2). The recurrence rate significantly increased according to the VI grade as follows: v0 (1.5%), v1 (29.6%), v2 (41.7%) and v3 (78.6%) (Table 2). By the univariate Cox analyses, age, macroscopic type, high-grade histology, depth of invasion, cancer stromal volume, tumor infiltrating pattern, VI grade, lymphatic invasion, nodal metastasis, resection margin status, pTNM stage, and AC, were raised as candidate predictors of recurrence (Table 3). We then narrowed down the variables to be entered in the multivariate analysis. Macroscopic type was significantly associated with depth of tumor invasion because type 0 means early cancer. High-grade histology, cancer stromal volume, and tumor infiltrating pattern was united in scirrhous pattern when GC satisfied histology (poorly differentiated adenocarcinoma (por), poorly cohesive type and/or signet-ring cell carcinoma (sig)), cancer stromal volume (sci) and tumor infiltration pattern (INFc) simultaneously. Lymphatic invasion results in nodal metastasis, which as well as depth of tumor invasion determines pTNM stage. Performance of AC was significantly correlated with higher pTNM Stage and VI grade (Table S2). Finally, age, scirrhous pattern, VI grade, resection margin status, and pTNM stage were subjected to the multivariate Cox analysis, and VI grade as well as pTNM stage was found to be an independent recurrence predictor with a statistical significance (Table 3).

**Table 2:** Recurrence rate according to the grade of venous invasion

		Grade of venous invasion				Total
		v0	v1	v2	v3	
Total	Recurrence (%)	1/65 (1.5%)	16/54 (29.6%)	10/24 (41.7%)	11/14 (78.6%)	38/157 (24.2%)
	RR (95%CI)	Reference	19.3 (3.6–113.3)	27.1 (5.0–162.3)	51.1 (11.1–289.9)	
	*p value		< 0.001	< 0.001	< 0.001	
	Median DTR (Range)	405	472.5 (42–2111)	351.5 (102–757)	206 (97–771)	369 (42–2111)
Without AC indication	Recurrence (%)	1/59 (1.7%)	2/29 (6.9%)	2/9 (22.2%)	2/5 (40.0%)	7/102 (6.9%)
	RR (95%CI)	Reference	4.07 (0.54–30.82)	13.1 (1.8–97.7)	23.6 (3.4–169.1)	
	*p value		0.252	0.044	0.014	
	Median DTR (Range)	405	354.5 (351–358)	220.5 (182–259)	130.5 (97–164)	259 (97–405)
With AC indication	Recurrence (%)	0/6 (0.0%)	14/25 (56.0%)	8/15 (53.3%)	9/9 (100.0%)	31/55 (56.4%)
	RR (95%CI)	Reference	N.C	N.C	N.C	
	*p value		0.021	0.046	< 0.001	
	Median DTR (Range)		644.5 (42–2111)	383 (102–757)	376 (131–771)	394 (42–2111)

*Statistical analysis was performed by Fisher’s exact test. *AC* adjuvant chemotherapy, *RR* relative risk, *CI* confidence interval, *DTR* days to recurrence, *N.C* not calculated, *v0* no venous invasion, *v1* 1–3 invasions/slide, *v2* 4–6 invasions/slide, *v3* no less than 7/slide. Filling type of venous invasion in macroscopically identifiable vein with a minor axis of ≥ 1 mm raised the grade of v1 or v2 by 1

**Table 3:** Recurrence predictors of gastric cancer by Cox regression analysis

Parameters	HR (95% CI)	*p value*
Univariate analysis		
Age	1.052 (1.017–1.088)	0.003
Sex (Male)	1.772 (0.812–3.867)	0.151
Location (Lower)	1.067 (0.556–2.044)	0.846
Surgery (TG)	1.196 (0.612–2.338)	0.601
Lymph node dissection (D2)	1.294 (0.683–2.454)	0.429
Synchronous multiple GCs (Yes)	1.025 (0.364–2.890)	0.963
Macroscopic type (3 + 4)	3.577 (1.883–6.796)	< 0.001
Depth of tumor invasion	2.827 (2.032–3.933)	< 0.001
Histology (por sig nec)	2.134 (1.076–4.229)	0.030
Cancer stromal volume (sci)	2.293 (1.203–4.371)	0.012
Tumor infiltration pattern (INFc)	1.731 (0.913–3.282)	0.093
*Scirrhous pattern	3.928 (2.077–7.428)	< 0.001
VI grade	2.967 (2.161–4.072)	< 0.001
Lymphatic invasion (Yes)	13.902 (4.270–45.262)	< 0.001
Nodal metastasis	2.404 (1.841–3.139)	< 0.001
Resection margin status (R1 + R2)	4.619 (1.919–11.120)	0.001
pTNM stage	4.152 (2.633–6.547)	< 0.001
Neoadjuvant chemotherapy (Yes)	2.295 (0.314–16.795)	0.413
Adjuvant chemotherapy (Yes)	4.651 (2.208–9.796)	< 0.001
Multivariate analysis		
Age	1.035 (0.998–1.073)	0.065
Scirrhous pattern	1.507 (0.729–3.115)	0.269
VI grade	2.119 (1.439–3.120)	< 0.001
Resection margin status	0.802 (0.315–2.040)	0.643
pTNM stage	2.577 (1.496–4.437)	0.001

*GC was evaluated as scirrhous pattern when it revealed histological high-grade of malignancy (por, poorly cohesive, and/or sig), abundant cancer stromal volume (sci) and diffuse tumor cell infiltration (INFc) simultaneously. 17 of 119 GCs without recurrence and 19 of 38 GCs with recurrence were evaluated as scirrhous pattern. *por* poorly differentiated adenocarcinoma, either solid or poorly cohesive type, *sig* signet-ring cell carcinoma, *nec* neuroendocrine carcinoma, *HR* hazard ratio, *CI* confidence interval, *TG* total gastrectomy, *GC* gastric cancer, *VI* venous invasion, p*TNM* pathological tumor node metastasis

We next investigated predictors of site-specific recurrence. VI grade and pTNM stage were both significant predictors of hematogenous, nodal, and peritoneal recurrences by the univariate Cox analyses. (Table 4) There were several candidate predictors of each site-specific recurrence, but given the small number of event in each site, prognostic significance of the VI grade was adjusted only with pTNM stage by the multivariate Cox analyses. VI grade was confirmed to be a significant predictor of hematogenous and peritoneal recurrence (*p* < 0.001 and *p* = 0.026, respectively) independent of pTNM stage but not of nodal recurrence (*p* = 0.316) (Table 4).

**Table 4:** Recurrence predictors of site-specific recurrence by Cox regression analysis

Parameters	Hematogenous recurrence (n = 17)		Nodal recurrence (n = 11)		Peritoneal recurrence (n = 14)	
	HR (95% CI)	*p* value	HR (95% CI)	*p* value	HR (95% CI)	*p* value
Univariate analysis						
Age	1.044 (0.994–1.096)	0.008	1.116 (1.034–1.203)	0.005	1.071 (1.009–1.136)	0.024
Sex (Male)	1.510 (0.492–4.633)	0.472	0.591 (0.180–1.938)	0.385	1.221 (0.383–3.897)	0.736
Location (Lower)	3.210 (0.922–11.173)	0.067	1.830 (0.485–6.901)	0.372	0.942 (0.327–2.715)	0.911
Surgery (TG)	0.310 (0.071–1.356)	0.120	0.890 (0.236–3.355)	0.863	1.276 (0.427–3.810)	0.663
Lymph node dissection (D2)	1.463 (0.557–3.845)	0.440	1.265 (0.386–4.146)	0.698	1.075 (0.376–3.070)	0.893
Synchronous multiple GCs (Yes)	1.188 (0.271–5.195)	0.819	0.841 (0.107–6.578)	0.869	0.646 (0.084–4.945)	0.674
Macroscopic type (3 + 4)	2.343 (0.865–6.347)	0.094	2.707 (0.790–9.277)	0.113	6.333 (2.184–18.363)	0.001
Depth of tumor invasion	2.232 (1.436–3.470)	< 0.001	6.074 (2.348–15.711)	< 0.001	3.985 (2.071–7.668)	< 0.001
Histology (por sig nec)	1.126 (0.778–1.628)	0.529	1.418 (0.851–2.364)	0.180	1.608 (0.981–2.636)	0.060
Cancer stromal volume (sci)	0.978 (0.340–2.817)	0.968	2.714 (0.827–8.903)	0.100	8.086 (2.254–29.011)	0.001
Tumor infiltration pattern (INFc)	0.753 (0.278–2.035)	0.575	3.838 (1.017–14.479)	0.047	8.689 (1.942–38.882)	0.005
VI grade	3.845 (2.331–6.343)	< 0.001	2.513 (1.402–4.505)	0.002	3.157 (1.846–5.397)	< 0.001
Lymphatic invasion (Yes)	18.491 (2.448–139.653)	0.005	88.559 (0.639–12265.7)	0.075	7.574 (1.691–33.931)	0.008
Nodal metastasis	2.407 (1.608–3.602)	< 0.001	4.057 (2.062–7.983)	< 0.001	2.444 (1.585–3.771)	< 0.001
Resection margin status (R1 + R2)	6.932 (2.252–21.332)	0.001	11.132 (2.899–42.745)	< 0.001	4.899 (1.072–22.396)	0.040
pTNM stage	3.638 (1.898–6.974)	< 0.001	16.017 (2.661–96.403)	0.002	7.631 (2.759–21.107)	< 0.001
Neoadjuvant chemotherapy (Yes)	4.697 (0.621–35.531)	0.134	0.049 (0.000- 3.3E + 09)	0.812	0.049 (0.000–2.474E + 8)	0.791
Adjuvant chemotherapy (Yes)	5.844 (1.880–18.165)	0.002	7.492 (1.551–36.194)	0.012	3.926 (1.310–11.765)	0.015
Multivariate analysis						
VI grade	3.145 (1.809–5.470)	< 0.001	1.495 (0.681–3.280)	0.316	2.090 (1.092–3.999)	0.026
pTNM stage	2.099 (1.023–4.309)	0.043	14.189 (2.275–88.511)	0.005	5.759 (1.978–16.769)	0.001

*HR* hazard ratio, *CI* confidence interval, *TG* total gastrectomy, *GC* gastric cancer, *por* poorly differentiated adenocarcinoma, either solid or poorly cohesive type, *sig* signet-ring cell carcinoma, *nec* neuroendocrine carcinoma, *VI* venous invasion, *pTNM* pathological tumor node metastasis

### Recurrence predictors of GC without AC indication

In Japan, AC is recommended to GC with pStages II/III except for pT1N2/N3 and pT3N0 (pStage IIA/IIB) based on the Adjuvant Chemotherapy Trial of TS-1 for Gastric Cancer (ACTS-GC) trial [8, 15, 16]. We then classified our patients in two tiers with and without AC indication and analyzed the prognostic impact of the VI grade separately. Clinicopathologic characteristics of each group are summarized in Table 5.

**Table 5:** Clinicopathologic characteristics of gastric cancer with and without indication for adjuvant chemotherapy

Parameters	Without AC indication			With AC indication		
	Recurrence		*p value*	Recurrence		*p value*
	Yes (n = 7)	No (n = 95)		Yes (n = 31)	No (n = 24)	
Age						
Median (range)	71 (70–86)	70 (42–92)	0.199	79 (42–96)	73 (55–84)	0.040
Sex						
Male/Female	7/0	63/32	0.095	23/8	18/6	1.000
Location						
Upper/Middle/Lower/extending in 2 or 3 regions	0/0/7/0	22/14/56/3	0.360	6/4/16/5	4/5/12/3	0.950
Surgery						
DG/TG/others	6/1/0	70/23/2	1.000	18/12/1	10/14/0	0.273
Lymph node dissection						
D1/D1+/D2	0/5/2	8/47/40	0.852	3/9/19	2/7/15	1.000
Synchronous multiple GCs						
Yes/No	2/5	9/86	0.165	2/29	4/20	0.387
Macroscopic type						
0/1/2/3/4/5	3/0/1/0/1/2	75/5/8/3/0/4	0.774	0/4/10/7/9/1	0/1/6/7/7/3	0.397
Histology						
pap tub1/tub2/muc/por sig nec	4/1/0/2	34/24/0/37	N.C	2/4/1/24	1/4/0/19	1.000
Depth of tumor invasion						
pT1/pT2/pT3	3/1/3	75/8/12	0.229	2/12/17	4/15/5	0.093
Cancer stromal volume						
med /int/sci/not described	0/4/2/1	3/43/10/39	0.947	0/14/17/0	2/11/9/2	0.551
Tumor infiltration pattern						
INFa/INFb/INFc/not described	0/5/2/0	3/46/18/28	1.000	1/11/19/0	0/8/15/1	1.000
VI grade						
v0/v1/v2/v3	1/2/2/2	58/27/7/3	0.064	0/14/8/9	6/11/7/0	0.012
Lymphatic invasion						
Yes/No	4/3	23/72	0.078	31/0	24/0	N.C
Nodal metastasis						
pN0/pN1/pN2	4/2/1	87/4/4	0.158	6/8/17	8/6/10	0.667
Resection margin status						
R0/R1/R2	7/0/0	94/1/0	1.000	25/5/1	23/1/0	0.591
pTNM stage						
I/II/III	3/4/0	79/16/0	0.026	0/6/25	0/8/16	0.350
Neoadjuvant chemotherapy						
Yes/No/unknown	0/7/0	0/83/12	N.C	1/28/2	1/22/1	1.000
Adjuvant chemotherapy						
Yes/No/unknown	4/3/0	9/74/12	0.008	19/7/5	20/3/1	0.299

*AC* adjuvant chemotherapy, *DG* distal gastrectomy, *TG* total gastrectomy, *GC* gastric cancer, *pap* papillary adenocarcinoma, *tub1* well-differentiated tubular adenocarcinoma, *tub2* moderately differentiated tubular adenocarcinoma, *muc* mucinous adenocarcinoma, *por* poorly differentiated adenocarcinoma, either solid or poorly cohesive type, *sig* signet-ring cell carcinoma, *nec* neuroendocrine carcinoma, *VI* venous invasion, p*TNM* pathological tumor node metastasis, *v0* no venous invasion, *v1* 1–3 invasions/slide, *v2* 4–6 invasions/slide, *v3* no less than 7/slide. Filling type of venous invasion in macroscopically identifiable vein with a minor axis of ≥ 1 mm raised the grade of v1 or v2 by 1, *N.C* not calculated

A total of 102 cases with stage I (n = 82) and a part of IIA (pT1N2 and pT3N0) (n = 20) were classified as GC without AC indication. There was no pT1N3 case. In this tier, recurrence was observed in 7 (6.9%) patients, consisting of 3 pStage I (1 pT2N0, and 2 pT1N1) and 4 pStage IIA (1 pT1N2 and 3 pT3N0). Local, nodal, peritoneal, and hematogenous recurrences were observed in 2, 0, 2, 4 patients, respectively. One patient with pStage IIA had local and peritoneal recurrences. The recurrence rate increased according to the grade of venous invasion as follows: v0 (1.7%), v1 (6.9%), v2 (22.2%) and v3 (40.0%) (Table 2). Recurrence rates of v2 and v3 were significantly higher than v0.

**Fig. 3:**
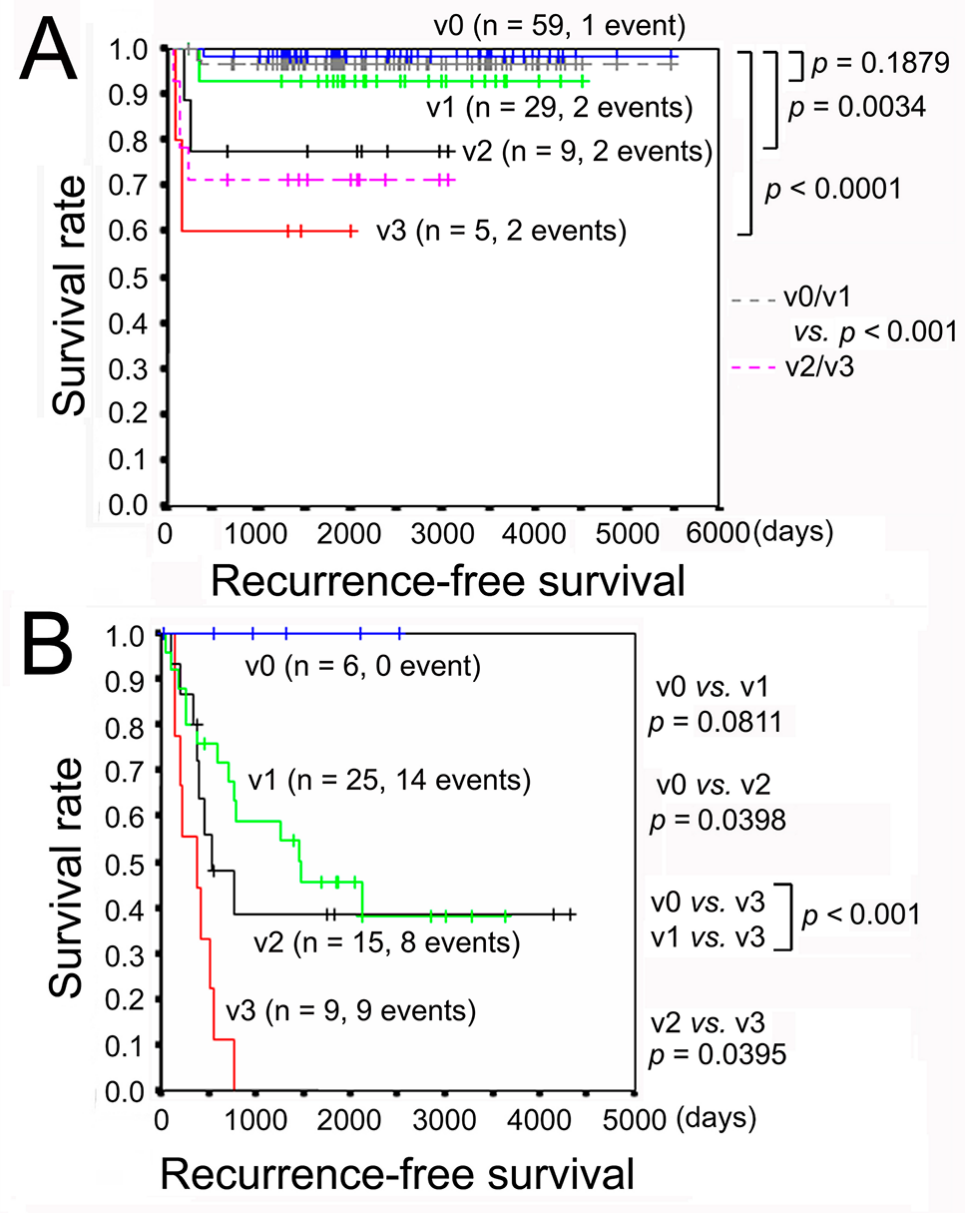
Recurrence-free survival of GC according to the grade of venous invasion. **a** GC without AC indication. **b** GC with AC indication. GC, gastric cancer; AC, adjuvant chemotherapy; v0, no venous invasion; v1, 1−3 invasions per glass slide; v2, 4−6 invasions per glass slide; and v3, ≥ 7 invasions per glass slide. Filling-type invasion in veins with a minor axis of ≥ 1 mm increased the grade by 1

Multivariate analysis was performed to adjust for confounding factors. Depth of tumor invasion, VI grade, lymphatic invasion, nodal metastasis, pTNM stage, and AC, were selected as candidate prognostic factors by the univariate analyses (Table 6). Lymphatic invasion results in nodal metastasis, which as well as depth of tumor invasion determines pTNM Stage. AC was performed more frequently in pStage IIA than in pStage I with a statistical significance (Table S3). Accordingly, VI grade and pTNM stage were subjected to the multivariate analysis, and only VI grade was found to be significant (Table 6). The Kaplan-Meier curves exhibited that RFS rates of v0 and v1 exceeded 90% even 10 years after surgery, while those of v2 and v3 fell below 80% by one year postoperatively (Fig. 3A).

**Table 6:** Recurrence predictors of gastric cancer with and without indication for adjuvant chemotherapy by Cox regression analysis

Parameters	Without AC indication (n = 102)		With AC indication (n = 55)	
	HR (95% CI)	*p* value	HR (95% CI)	*p* value
Univariate analysis				
Age	1.054 (0.974–1.141)	0.193	1.042 (1.005–1.081)	0.026
Sex (Male)	N.C		1.348 (0.599–3.033)	0.471
Location (Lower)	N.C		0.816 (0.402–1.656)	0.574
Surgery (TG)	0.541 (0.065–4.494)	0.570	0.722 (0.346–1.504)	0.384
Lymph node dissection (D2)	0.553 (0.107–2.853)	0.480	0.760 (0.368–1.570)	0.458
Synchronous multiple GCs (Yes)	3.551 (0.688–18.330)	0.130	0.455 (0.108–1.919)	0.284
Macroscopic type (3 + 4)	4.844 (0.582–40.310)	0.144	0.763 (0.376–1.548)	0.453
Depth of tumor invasion	2.382 (1.084–5.230)	0.031	1.678 (0.945–2.981)	0.077
Histology (por sig nec)	0.652 (0.127–3.361)	0.609	0.736 (0.316–1.716)	0.478
Cancer stromal volume (sci)	2.144 (0.393–11.710)	0.379	1.090 (0.537–2.214)	0.811
Tumor infiltration pattern (INFc)	1.083 (0.210–5.582)	0.924	0.694 (0.336–1.432)	0.322
VI grade	3.199 (1.624–6.302)	0.001	2.319 (1.497–3.593)	< 0.001
Lymphatic invasion (Yes)	3.865 (0.865–17.277)	0.077	N.C	
Nodal metastasis	2.664 (1.106–6.415)	0.029	1.370 (0.884–2.125)	0.159
Resection margin status (R1 + R2)	N.C		2.047 (0.830–5.046)	0.120
pTNM stage	5.984 (1.338–26.767)	0.019	1.577 (0.646–3.847)	0.317
Neoadjuvant chemotherapy (Yes)	N.C		0.835 (0.113–6.157)	0.860
Adjuvant chemotherapy (Yes)	9.875 (2.200–44.334)	0.003	0.315 (0.130–0.760)	0.010
Multivariate analysis				
Age	N.A		1.011 (0.966–1.059)	0.631
Depth of tumor invasion	N.A		1.581 (0.804–3.107)	0.184
VI grade	2.688 (1.240–5.825)	0.012	1.918 (1.137–3.237)	0.015
pTNM stage	2.273 (0.413–12.494)	0.345	N.A	
Adjuvant chemotherapy (Yes)	N.A		0.387 (0.137–1.091)	0.073

*AC* adjuvant chemotherapy, *HR* hazard ratio, *CI* confidence interval, *TG* total gastrectomy, *GC* gastric cancer, *pap* papillary adenocarcinoma, *por* poorly differentiated adenocarcinoma, either solid or poorly cohesive type, *sig* signet-ring cell carcinoma, *nec* neuroendocrine carcinoma, *VI* venous invasion, *pTNM* pathological tumor node metastasis, *N.C* not calculated, *N.A* not applicable

### Recurrence predictors for GC with AC indication

A total of 55 cases with pStages IIA (pT2N1), IIB, and III were classified as indication for AC, although 10 cases had not been actually administered AC at the surgeons’ discretion. In this tier, 31 (56.4%) patients suffered from recurrence, which was not observed in v0 but occurred in more than 50% of cases with v1, v2 and v3 (Table 2). Local, non-regional nodal, peritoneal, and hematogenous recurrences were observed in 0, 11, 12, 13 patients, respectively.

Multivariate analysis was performed to adjust for confounding factors. Age, depth of tumor invasion, VI grade, and AC were selected as candidate predictors by the univariate analysis. AC was included in the multivariate analysis, because it was statistically independent of pTNM Stage (Table S3). As a result, VI grade was revealed to be an independent predictor of recurrence (*p* = 0.015) (Table 6). In addition, AC was found to marginally decrease recurrence risk (*p* = 0.073).

The Kaplan-Meier curves demonstrated that RFS rate of v0 was 0% but RFS rate fell below 80% within one year postoperatively when VI was positive. The RFS rate of v1 was less than 60% by post-operative day (POD) 1000 and those of v2 and v3 fell below 50% approximately after POD 500 (Fig. 3B).

### Postoperative recurrence and histological subtype

**Fig. 4:**
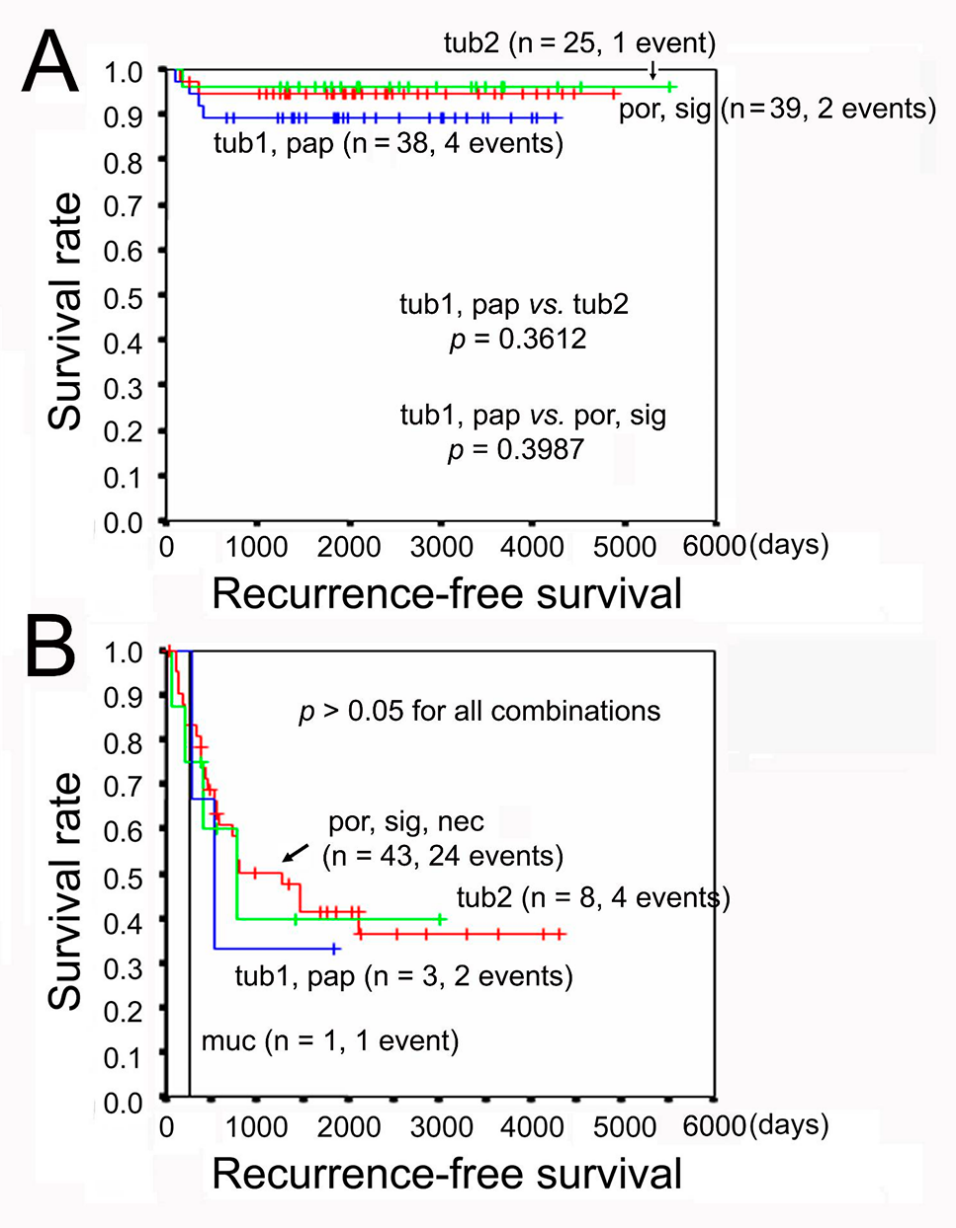
Recurrence-free survival of GC according to the histological subtype. **a** GC without AC indication. **b** GC with AC indication. GC, gastric cancer; AC, adjuvant chemotherapy; tub1, well-differentiated tubular adenocarcinoma; tub2, moderately differentiated tubular adenocarcinoma, pap, papillary adenocarcinoma; muc, mucinous adenocarcinoma; por, poorly differentiated adenocarcinoma, either solid or poorly cohesive type; sig, signet-ring cell carcinoma; nec, neuroendocrine carcinoma. There was no case of nec in the tier without AC indication, while there were two cases of nec in the tier with AC indication

To our surprise, histological subtype or differentiation grade per se was not demonstrated to be a postoperative predictor in any of the above analyses. The RFS curves of GC with and without AC indication also demonstrated no significant differences among histological subtypes in each tier (Fig. 4).

## Discussion

In the UICC TNM staging system (8th ed.), V1 and V2 are defined as microscopic and macroscopic VI, respectively [4]. By contrast, the latest JCGC (15th ed.) classifies VI as V0 (none), V1a (mild), V1b (moderate), and V1c (severe) based on the pathologist’s subjectivity, without distinction between microscopic and macroscopic VI [5]. In this study, our VI grading system integrated VI number per glass slide and size of the invaded vein on the assumption that the VI number and size of the invaded veins would both positively correlate with metastatic potential. In similar studies of colorectal cancer (CRC), VI was further subclassified by other factors such as morphology and location [10]. To be more specific, VI was morphologically classified into the filling type, the floating type in which tumor cells did not adhere to the vein and floated in the lumen, and the infiltrating type in which tumor cells were seen infiltrating the wall of the vessel. However, these types often coexist and the survival impact between the filling type and nonfilling type was not significant. In addition, VI was subclassified into intramural and extramural in CRC, but we considered that there may be little reason to distinguish them because they are connected.

Nakanishi et al. investigated postoperative courses of 132 patients who had undergone curative gastrectomy for advanced GC (pT2−4) and found that the VI grade was an independent prognostic factor for hematogenous recurrence but not for peritoneal and nodal metastases [17]. Takeuchi et al. analyzed pT1N + GC patients (n = 97) who underwent radical gastrectomy and found that VI was an independent risk factor for recurrence [18]. Nine of 12 recurrences in their study were hematogenous. Nishibeppu et al. analyzed 97 patients who underwent AC after curative gastrectomy for pStage III GC and found that VI was an independent predictive factor of shorter RFS and OS [19]. In contrast, Yu et al. analyzed postoperative prognosis of 253 patients with stage IB GC and did not find a significant association between VI and recurrence [20]. Eleven of the 14 recurrences in their study were hematogenous. Zhu et al. analyzed 249 patients with T4 GC who underwent curative resection and did not find a significant association between VI and recurrence, 26.7%, 24.4%, and 62.2% of which were hematogenous, nodal, and peritoneal metastases, respectively [21]. All these studies recorded VI as positive or negative, which was evaluated by hematoxylin and eosin (HE) staining [20] or staining not specified [17–19, 21]. Fukuda et al. also did not find a significant prognostic impact of VI in 71 patients with T4 GC after curative resection [22]. In their study, VI was classified in either v0/v1 or v2/v3 by the JCGC (3rd English ed.), and the staining method was not specified. Here, v0, v1, v2, and v3 corresponded to V0, V1a, V1b, and V1c in the JCGC (15th ed.), respectively. Araki et al. investigated prognostic impact of VI in 130 GC patients staged T2N0 and T3N0 by the UICC TNM staging system (8th ed.) and found that moderate or marked venous invasion (v2 or v3) was an only significant predictor of recurrence and cancer-related death [23]. In their study, VI was graded by the EVG staining as follows: v0, VI was not found on any slide examined; v1, one or two sites of VI throughout all eight slides examined; v2, intermediate level between v1 and v3; v3, one or more sites of VI on every slide examined. Recurrence occurred in 12 patients. Among them, 7, 2, 2, and 1 were hematogenous, nodal, peritoneal, and locoregional, respectively.

Most studies evaluated VI as positive or negative, and the staining method was not specified. In contrast, we graded VI objectively in a simple way by the EVG staining and demonstrated that postoperative recurrence significantly increased according to the VI grade (Table 2). The VI grade as well as pTNM stage was an independent recurrence predictor with a statistical significance, consistent with the previous studies [17–19, 23]. Although VI was not associated with postoperative recurrence in some studies which analyzed stage I and T4 GC [20–22], we speculate that it may be due to the intrinsic excellent and dismal prognoses of stage I and T4 GCs, respectively. In fact, many studies concluded that the lymphatic/vascular invasions are not directly related to patients’ prognosis with stage I GC [24]. The low sensitivity for detecting VI may also underlie the results when VI was assessed by the HE staining.

Our site-specific recurrence analyses revealed that VI grade was an independent predictor of hematogenous and peritoneal recurrences but not of nodal recurrence. VI has been reported to be associated with hematogenous metastasis as with the present study [17, 18, 23]. Furthermore, Nakanishi et al. reported that VI was not associated with nodal recurrence consistent with our results [17]. Unlike our results, they reported that VI was not associated with peritoneal recurrence, which was significantly associated with lymph node metastasis and differentiation grade [17]. Zhu et al. also reported that not VI but pN stage was significantly associated with recurrence of T4 GC after curative resection, 62.2% of which was peritoneal implanting [21]. Studies by Nakanishi et al. and Zhu et al. analyzed advanced GC staged pT2−4 and we speculate that inclusion of pT1 comprising more than half of our subjects may explain the inconsistent results with their studies.

AC is performed to decrease the risk of recurrence. The SWOG INT-0116 trial reported in 2001 [25] exhibited positive results of AC with fluorouracil plus leucovorin for GC with stages IB to III by the UICC TNM staging system (8th ed.). In Japan, AC has been recommended to GC with stages II/III except for T1N2/N3 and T3N0 since 2007, based on the ACTS-GC trial that analyzed GC which has undergone D2 gastrectomy [8]. In other words, GC with stages I and a part of II (T1N2/N3 and T3N0) is not an indication for AC. We then proceeded to analyzing patients with and without AC indication as per the ACTS-GC trial separately. Postoperative recurrence had been observed in 7/102 (6.9%) patients without AC indication and 31/55 (56.4%) patients with AC indication.

For the tier without AC indication, VI grade was the only significant predictor of postoperative recurrence (Table 6). RFS deteriorated according to the VI grade, while v0 and v1 exhibited similar RFS curves (Fig. 3A). The RFS rate of the patients with the VI grade of v0 + v1 exceeded 95% even after 5 years after surgery, while that of the patients with v2 + v3 was 71.4% on POD 259. These results suggest that AC may be considered for pStages I and IIA (T1N2/N3 and T3N0) with the VI grade of no less than v2.

For the tier with AC indication, the VI grade was the only significant predictor of postoperative recurrence and AC was marginally effective in decreasing recurrence (Table 6). RFS deteriorated according to the VI grade (Fig. 3B). The VI grade v0 was observed only in 6 (10.9%) of 55 patients in this tier, but recurrence occurred in none of them during follow-up. In contrast, the RFS rate fell below 80% within one year postoperatively when VI was positive. These results support the validity of the present AC indication.

According to the ACTS-GC study, S-1 monotherapy could significantly reduce nodal and peritoneal metastases but could hardly control the hematogenous metastasis [8]. Recently, several promising studies have reported the efficacy of combination chemotherapy as a more intensive adjuvant therapy. The CLASSIC study, analyzing GC that underwent D2 gastrectomy and staged II and III except for T1N2 and T3N0 by the UICC TNM staging system (8th ed.), reported that capecitabin plus oxaliplatin could well control distant metastasis but could not control peritoneal and lymph nodal metastases [26]. The JACCRO GC-07 trial, analyzing stage III GC that underwent D2 resection based on the JCGC (3rd English ed.), reported that postoperative S-1 plus docetaxel significantly decreased hematogenous and nodal metastases than S-1 alone [27]. These regimens may be considered when high-grade VI (v2 and v3) was confirmed in the postoperative pathological examination.

During the present study, histological subtype or differentiation grade per se was not a recurrence predictor unlike CRC and pancreatic cancer [10, 28]. Pathologists evaluate histological grading based on the least differentiation grade in CRC and pancreatic cancer but on the predominant histology in GC. We therefore attempted multivariate analysis with the least differentiated grade, which was not significant again (data not shown). The result may be explained by readily dedifferentiation of GC resulting in an admixture of various differentiation grades and histological subtypes in one tumor.

Our study has some limitations. First, this study was retrospective and was performed at a single institution. Therefore, numbers of event were small, especially in the tier without AC indication. Second, the time span ranged over 14 years during which there have been changes in surgical techniques, such as spleen preservation and laparoscopic surgery. Indications and regimens of AC have also changed. Such a background should be remembered when interpreting our data.

## Conclusions

In the present study, we demonstrated that VI grade was an independent predictor of postoperative GC recurrence irrespective of the AC indication based on the ACTS-GC study. This VI grading system could be applied in future studies of adjuvant therapy in GC presently deemed without AC indication in Japan.

## Electronic supplementary material

Below is the link to the electronic supplementary material.

**Table S1** Statistics used for comparing GC between with and without recurrence

**Table S2** Administration of AC according to pTNM stage and VI grade

**Table S3** Administration of AC according to pTNM stage in GC without and with AC indication

## Data Availability

All data generated or analyzed in this study are included in this published article and additional files.

## Abbreviations

GC: Gastric cancer
VI: Venous invasion
UICC: Union for International Cancer Control
TNM: Tumor, node, metastasis
JCGC: Japanese Classification of Gastric Carcinoma
EVG: Elastica van Gieson
AC: Adjuvant chemotherapy
p: Pathological
S-1: Tegafur/gimeracil/oteracil
WHO: World Health Organization
RFS: Recurrence-free survival
OS: Overall survival
ACTS-GC: Adjuvant Chemotherapy Trial of TS-1 for Gastric Cancer
POD: Post-operative day
CRC: Colorectal cancer
HE: Hematoxylin and eosin
