# Supplementary material for: Risk stratification for predicting postoperative recurrence of gastric cancer by grade of venous invasion
Source: BMC Gastroenterol. 2023 May 30;23:189. doi: 10.1186/s12876-023-02825-0 (PMC10228042; doi:10.1186/s12876-023-02825-0)
Supplement: Supplementary file 1 — Table S1 Statistics used for comparing GC between with and without recurrence [file 12876_2023_2825_MOESM1_ESM.pdf]

Table S1 Statistics used for comparing GC between with and without recurrence

Parameters	Statistics
Age	Mann-Whitney *U* test
Sex	Fisher's exact test
Location	chi-square test with Yates' correction: Upper *vs.* Middle *vs* . Lower
Surgery	Fisher's exact test
Lymph node dissection	chi-square test with Yates' correction
Synchronous multiple GCs	Fisher's exact test
Macroscopic type	chi-square test with Yates' correction: 0 *vs* .1+2 *vs* . 3+4
Histology	chi-square test with Yates' correction
Depth of tumor invasion	Mann-Whitney *U* test
Cancer stromal volume	chi-square test with Yates' correction
Tumor infiltration pattern	chi-square test with Yates' correction
VI grade	Mann-Whitney *U* test
Lymphatic invasion	Fisher's exact test
Nodal metastasis	Mann-Whitney *U* test
Resection margin status	Mann-Whitney *U* test
pTNM stage	Mann-Whitney *U* test
Neoadjuvant chemotherapy	Fisher's exact test
Adjuvant chemotherapy	Fisher's exact test

VI venous invasion, *GC* gastric cancer.
