# Supplementary material for: Risk stratification for predicting postoperative recurrence of gastric cancer by grade of venous invasion
Source: BMC Gastroenterol. 2023 May 30;23:189. doi: 10.1186/s12876-023-02825-0 (PMC10228042; doi:10.1186/s12876-023-02825-0)
Supplement: Supplementary file 2 — Table S2 Administration of AC according to pTNM stage and VI grade [file 12876_2023_2825_MOESM2_ESM.pdf]

Table S2 Administration of AC according to pTNM stage and VI grade

	AC		*p value
	Administered	Not administered	
pTNM stage			
I	3	68	< 0.001
II	19	12	
III	30	7	
VI grade			
v0	5	52	<0.001
v1	23	22	
v2	16	7	
v3	8	6	

* Mann-Whitney *U* test. *AC* adjuvant chemotherapy, *VI* venous invasion, *pTNM* pathological tumor node metastasis, *v0* no venous invasion, *v1* 1-3 invasions/slide, *v2* 4-6 invasions/slide, *v3* no less than 7/slide. Filling type of venous invasion in macroscopically identifiable vein with a minor axis of ≥ 1 mm raised the grade of v1 or v2 by 1.
