# Supplementary material for: Risk stratification for predicting postoperative recurrence of gastric cancer by grade of venous invasion
Source: BMC Gastroenterol. 2023 May 30;23:189. doi: 10.1186/s12876-023-02825-0 (PMC10228042; doi:10.1186/s12876-023-02825-0)
Supplement: Supplementary file 3 — Table S3 Administration of AC according to pTNM stage in GC without and with AC indication [file 12876_2023_2825_MOESM3_ESM.pdf]

Table S3 Administration of AC according to pTNM stage in GC without and with AC indication

	Without AC indication			With AC indication		
	AC		*p value	AC		**p value
	Administered	Not administered		Administered	Not administered	
pTNM stage						
I	3	68	< 0.001	N.A	N.A	
IIA	10	9		1	1	
IIB	0	0		8	2	0.585
III	N.A	N.A		30	7	

*Fisher's exact test. **Mann-Whitney *U* test. *AC* adjuvant chemotherapy, *VI* venous invasion, *pTNM* pathological tumor node metastasis, *N.A* not applicable.
